# The association between smoking or passive smoking and cardiovascular diseases using a Bayesian hierarchical model: based on the 2008-2013 Korea Community Health Survey

**DOI:** 10.4178/epih.e2017026

**Published:** 2017-06-22

**Authors:** Whanhee Lee, Sung-Hee Hwang, Hayoung Choi, Ho Kim

**Affiliations:** Graduate School of Public Health, Seoul National University, Seoul, Korea

**Keywords:** Smoking, Passive smoking, Cardiovascular diseases, Korea Community Health Survey, Korea

## Abstract

**OBJECTIVES:**

Smoking and passive smoking have been extensively reported as risk factors of cardiovascular morbidity and mortality. Despite the biological mechanisms underlying the impact of hazardous chemical substances contained in tobacco in cardiovascular diseases (CVD), studies investigating the association between smoking and passive smoking with morbidity are at an inchoate stage in Korea. Therefore, this study aimed to estimate the risks of smoking and passive smoking on cardiovascular morbidity at the national and regional levels.

**METHODS:**

This study calculated sex-standardized and age-standardized prevalence of CVD and smoking indices in 253 community health centers (si/gun/gu) in Korea using the 2008-2013 Korea Community Health Survey data. Furthermore, a Bayesian hierarchical model was used to estimate the association of smoking and passive smoking with the prevalence of CVD from the national and regional community health centers.

**RESULTS:**

At the national level, smoking was significantly associated with stroke (relative risk [RR], 1.060) and hypertension (RR, 1.016) prevalence, whilst passive smoking at home and work were also significantly associated with prevalence of stroke (RR, 1.037/1.013), angina (RR, 1.016/1.006), and hypertension (RR, 1.010/1.004). Furthermore, the effects of smoking and passive smoking were greater in urban-industrial areas than in rural areas.

**CONCLUSIONS:**

The findings of this study would provide grounds for national policies that limit smoking and passive smoking, as well as regionally serve as the basis for region-specific healthcare policies in populations with high CVD vulnerability.

## INTRODUCTION

Smoking and passive smoking have extensively been documented as risk factors of morbidity and mortality by a number of diseases [1-4]. Among more than 4,000 types of chemical substances contained in a cigarette, polycyclic aromatic hydrocarbons and oxidizing gases cause cardiac toxicity [1], having adverse effects on various cardiovascular diseases (CVD), including coronary artery disease, ischemic stroke, non-traumatic subarachnoid hemorrhage [5,6].

The World Health Organization reported that CVD-related death (1.69 million) is the leading cause of smoking-related death. In a study on 360 thousand US men, smoking has been identified as a key risk factor for lung cancer, chronic heart disease, and stroke [3]. In addition, a Norwegian study revealed that smokers are 2.74 times more likely to have a stroke and 6.74 times more likely to die from it than are non-smokers [4]. Similar to the effects of smoking, passive smoking has also been reported to be the cause of respiratory and CVD, various cancers, and premature deaths in non-smoking adults [7]. Other studies have suggested that secondhand smoking in non-smokers increases their risk of developing a heart disease by 25 to 30% [8-10].

In South Korea (hereafter Korea), several studies have reported that smoking increases the risk of CVD-related death in adults [11-13]. As of 2012, the number of smoking-related deaths was 4,148 for stroke and 3,858 for ischemic heart disease, which are ranked the second and third highest causes of death by an illness respectively [14]. Furthermore, it also has been reported that 26.7% of all CVD-related deaths in men and women adults in Korea are caused by smoking [13]. Although adult smoking rate in Korea has consistently been on a decline since 1998 [15], past smoking history may have an impact on death [11]. Moreover, medical expenses and patient population related to hypertension, heart disease, and cerebrovascular disease are continuously on the rise [16], suggesting that smoking is a health hazard in terms of CVD and mortality requiring much attention.

As shown here, though there have been much research investigating the association between smoking and death by CVD in Korea, studies on smoking and morbidity is relatively lacking. Hence, this study aimed at quantitatively analyzing the association between smoking and the prevalence of various CVD (i.e., hypertension, stroke, myocardial infarction, angina), and to evaluate the relative risk (RR) of smoking on the prevalence of CVD using data from a large-scale cross-sectional study with more than 1.5 million participants. We also examined the effects of passive smoking on the prevalence of CVD.

## MATERIALS AND METHODS

### Data

Data from the 2008-2013 Korea Community Health Survey (KCHS) involving 253 community health centers were used for this study [17]. The KCHS was conducted by the Korea Centers for Disease Control and Prevention, and data collected from adults aged 19 years or older via interviews. The sample was extracted from an average of 900 adults per community (si/gun/gu) based on the type of housing within each dong/eup/myeon. The primary sample region was obtained using a probability proportional to size systematic sampling, after which the secondary sample families were selected [18]. A total of 1,567,930 people were included in the KCHS data used in this study (2008: 200,800; 2009: 220,258; 2010: 230,712; 2011: 229,229; 2012: 229,226; 2013: 228,781), and the participants were randomly extracted every year. Using this data, we calculated the prevalence of 4 CVD (hypertension in adults ≥ 30 years, stroke in adults ≥ 50 years, myocardial infarction in adults ≥ 40 years, angina in adults ≥ 40 years), smoking-related regional parameters (current smoking rate, passive smoking rate at home, and passive smoking rate at work), body mass index (BMI), and monthly drinking rate by community (si/gun/gu), after reflecting gender/age distributions based on the year of study. The prevalence of CVD was measured, based on a self-reported response regarding a physician’s diagnosis, whilst BMI and monthly drinking rates were also measured with self-reported data.

### Statistical analysis

A generalized linear mixed model (GLMM) was used to analyze the association among regional and national smoking indices and prevalence of CVD that were repeatedly measured from 2008-2013. The prevalence of 4 CVD (hypertension in adults ≥ 30 years, stroke in adults ≥ 50 years, myocardial infarction in adults ≥ 40 years, and angina in adults ≥ 40 years) was set as the response variables. A Shapiro-Wilk test revealed that the prevalence of all 4 diseases did not follow a normal distribution, but instead showed a gamma distribution. Hence, a GLMM that assumes gamma distribution for the response variables was used. A log function was used as the link function. Mean BMI and monthly drinking rate per community (si/gun/gu) for 2008-2013, were set as the respective confounding variables and covariate, and a linear adjustment was made for annual effects to consider the effects of time. Smoking indices (current smoking rate, passive smoking rate at home, passive smoking rate at work) were used as the response variables, and we predicted associations with response variables with equal confounding variables and covariates.

Furthermore, a Bayesian hierarchical model was used with a GLMM to measure the effects of smoking indices of each community (si/gun/gu) and adjust for the correlations according to distance. The associations between community (si/gun/gu)-specific smoking indices and CVD were examined with random intercept and random slope, and the prior distributions of random intercept and random slope were assumed to follow a normal distribution with mean zero and an assumption that the inverse of variance follows a gamma distribution (parameter= 1, 5*10^-5^). Spatial correlation was adjusted for, with random intercept using the Besag-York-Mollie method [19], and all random intercepts and random slopes were assumed to be independent. Based on existing studies, the prior distribution of fixed effects was assumed a multivariate normal, which assumes variance to be a Gaussian Markov random field [20], and the posterior credibility interval (CI) was estimated based on the prior distribution. All analyses were performed using the R and R version 3.2.2 (https://cran.r-project.org/bin/windows/base/old/3.2.2/) for integrated nested Laplace approximation (INLA) [21]. More detailed formula and prior conditions are reported in Appendix 1.

## RESULTS

Table 1 shows the descriptive statistics for the variables used in this study. Hypertension over 30 years was the most prevalent (18.2%) throughout the 6 years of study (2008-2013), while angina over 40 years was the least prevalent (1.9%). The distributions of prevalence varied widely across the country. The average smoking rate as an interest variable during 6 years was 25.2% (14.9 to 33.4%), with an average passive smoking rate at home of 11.8% (2.3 to 37.3%) and an average passive smoking rate at work of 29.2% (6.0 to 76.7%).

Table 2 shows the top and bottom 5 community health centers in terms of the prevalence of CVD and smoking indices (6-year average). Hypertension over 30 years was generally prevalent in the Gangwon province (21.72 to 22.35%) and less prevalent in community health centers in the Gyeongsang province and Jeolla province (14.03 to 14.72%). Stroke over 50 years was generally prevalent in community health centers in the North Gyeongsang province (4.68 to 5.33%) and less prevalent in the South Jeolla province (1.93 to 2.12%). Myocardial infarction over 40 years tended to be prevalent in community health centers in Seoul and the Gyeonggi province (2.12 to 2.25%) and less prevalent in community health centers in North Jeolla province (0.78 to 0.83%). Angina over 40 years tended to be prevalent in community health centers in Busan (2.88 to 3.25%) and less prevalent in the North Jeolla province (1.03 to 1.08%). In terms of smoking parameters, smoking rate did not vary by region. Community health centers in Jeju Island showed higher passive smoking rates at home (19.88 to 21.80%) while community health centers in Ulsan showed higher passive smoking rates at work (40.93 to 41.70%).

The national average RR of smoking indices on the prevalence of CVD is shown in Table 3, whilst the regional average (RR) for top and bottom 5 regions is shown in Table 4. All RR shown in Tables 3 and 4 represent RR when smoking parameters increase by 5%. As shown in Table 3, smoking rate was most highly associated with stroke (RR, 1.060; 95% CI, 1.022 to 1.100), and it was also significantly associated with hypertension (RR, 1.016; 95% CI, 1.004 to 1.029). Passive smoking at home was most strongly associated with a rise in the prevalence of stroke (RR, 1.037; 95% CI, 1.023 to 1.051), and its RR for hypertension, stroke, and angina (but not myocardial infarction) was statistically significant (RR, 1.010 to 1.037). Passive smoking at work had the greatest effect on stroke (RR, 1.013; 95% CI, 1.007 to 1.019) and also had a significant effect on hypertension (RR, 1.004; 95% CI, 1.002 to 1.006).

**Figure 1.**
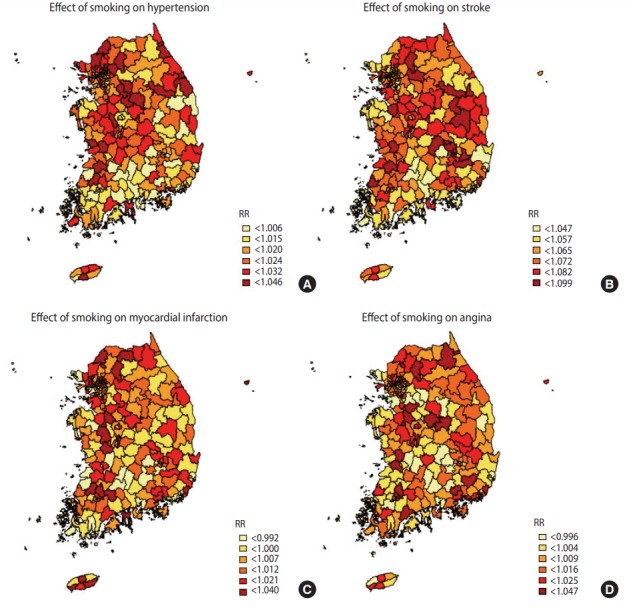
Geographical distribution of relative risks (RR). The associations between 4 cardiovascular diseases (A) hypertension over 30 years, (B) stoke over 50 years, (C) myocardial infarction over 40 years, and (D) angina over 40 years and smoking.

**Figure 2.**
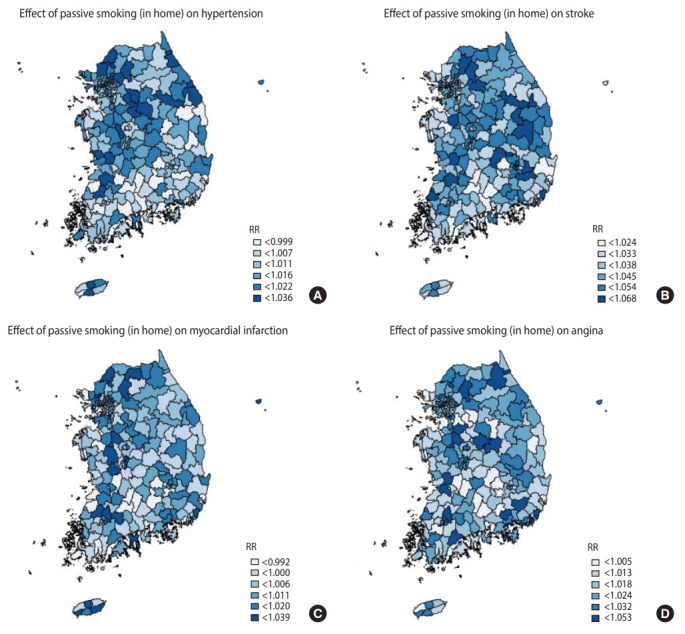
Geographical distribution of relative risks (RR). The associations between 4 cardiovascular diseases (A) hypertension over 30 years, (B) stoke over 50 years, (C) myocardial infarction over 40 years, and (D) angina over 40 years and passive smoking at home.

**Figure 3.**
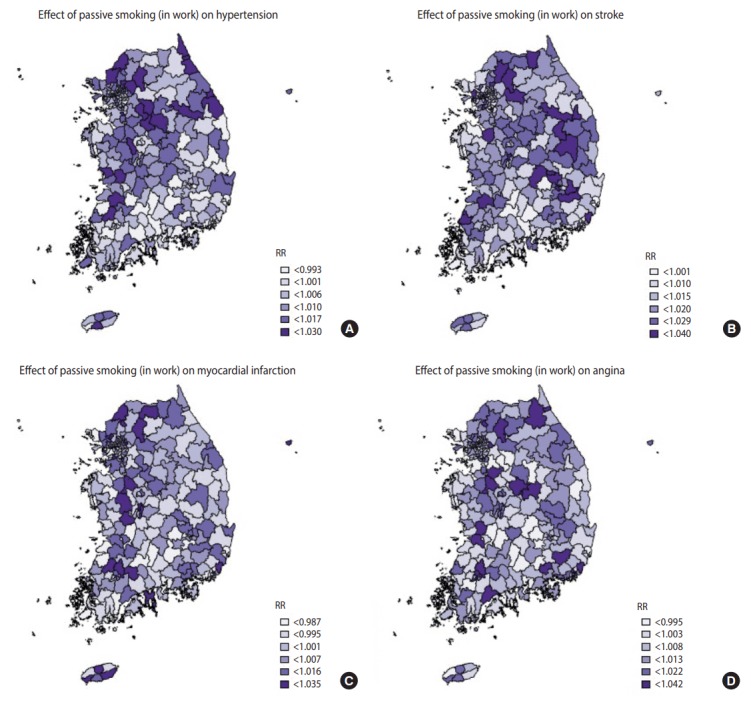
Geographical distribution of relative risks (RR). The associations between 4 cardiovascular diseases (A) hypertension over 30 years, (B) stoke over 50 years, (C) myocardial infarction over 40 years, and (D) angina over 40 years and passive smoking at work.

Table 4 shows the top and bottom three community health centers in terms of the RRs of smoking indices, whilst Figures 1-3 are national maps showing the effects of community-specific smoking rate and passive smoking rate at home and work on the prevalence of CVD. RR of smoking rate for hypertension was high in community health centers in Gyeonggi and Gangwon provinces (RR, 1.042 to 1.046) and low in community health centers in North Jeolla and Gyeonsang provinces (RR, 0.985 to 0.996). Furthermore, smoking was strongly associated with stroke in community health centers in North Gyeongsang province (RR, 1.095 to 1.099) but weakly associated with stroke in community health centers in Busan and Ulsan (RR, 1.033 to 1.034). The RR of smoking for myocardial infarction was high in community health centers in Daejeon (RR, 1.029 to 1.029), but there were no regional differences in terms of low RR (RR, 0.973 to 0.983). The effects of smoking on angina was high in the Gyeonggi region (RR, 1.040 to 1.035) and low in the southern region of the Korean peninsula (North Gyeonsang, North Jeolla, and Jeju Island) (RR, 0.975 to 0.989). The distributions of RR of passive smoking at home and work were similar to those of smoking, and the community rankings were similar as well. Exposure to passive smoking at home (RR, 1.003 to 1.037) had greater impact on all 4 CVD than did exposure to passive smoking at work (RR, 0.998 to 1.013). In addition, the RR of all smoking indices tended to be higher in the Seoul metropolitan area (Figures 1-3).

## DISCUSSION

This study analyzed the associations between regional smoking indices (smoking rate and passive smoking rate at home and work) and 4 CVD (hypertension in adults ≥ 30 years, stroke in adults ≥ 50 years, myocardial infarction in adults ≥ 40 years, angina in adults ≥ 40 years) with the KCHS data using a Bayesian hierarchical model. The results showed that smoking rate was significantly associated with stroke (RR, 1.060) and hypertension (RR, 1.016), and that passive smoking was significantly associated with stroke (RR, 1.013 to 1.037), angina (RR, 1.006 to 1.016), and hypertension (RR, 1.004 to 1.010) at the national level. Furthermore, exposure to passive smoking at home (RR, 1.003 to 1.037) had a greater impact on CVD than did exposure to passive smoking at work (RR, 0.998 to 1.013).

In addition to the national-level analysis, this study also analyzed the effects of smoking regionally in 253 regions, based on the assumption that specific regional features (geographical location, environment, income, population matrix) may have varying effects on the prevalence of CVD and may alter the effects of smoking on CVD. Our findings showed that RR of smoking varied across regions, suggesting that the effects of smoking on CVD varied across regions. The effects of smoking and passive smoking on all CVD were high in urban-industrial regions.

Nicotine, the primary ingredient of tobacco, is a sympathetic stimulant that acts on the central and peripheral nervous systems to stimulate the release of catecholamine and other neurotransmitters. It leads to cardiovascular consequences, such as elevating the heart rate, blood pressure, and cardiac output. Furthermore, it leads to a rise in low-density lipoprotein and decrease in high-density lipoprotein by mobilizing free fatty acids, thereby intensifying vasoconstriction and accelerating the progression of blood epithelial cell injury and atherosclerosis [1]. Carbon monoxide that is inhaled through smoking and passive smoking binds with hemoglobin to induce hypoxia, which increases the number of red blood cells and subsequently, blood viscosity thereby, having a direct impact on thrombosis and atherosclerosis [22]. Through these actions, smoking not only causes coronary artery diseases but also induces structural damage to arterial walls, and has also been associated with ischemic stroke caused by atherosclerosis and nontraumatic subarachnoid hemorrhage caused by formation and rupture of aneurysm [23].

This study presents data that support these biological causes. Although smoking indices were not statistically significantly associated with myocardial infarction and angina, the RR of smoking was above 1 for all CVDs at the national level (RR, 1.004 to 1.010). These results are in line with those reported by US and Norwegian studies on the relationship between smoking and heart diseases [3,4]. According to a foreign 20-year retrospective study, the greater the degree of exposure to passive smoking (cotinine concentration) was, the greater the risk for coronary artery disease and stroke [24]. Another study involving experimental and control groups revealed that the risk for myocardial infarction increases by 68% among passive smokers [25]. Furthermore, a meta-analysis of studies in 6 countries found that the RR for CVDs was between 1.2 and 1.3 [26]. In one study, passive smoking was found to be more of a health-hazard than direct smoking because smoke from the tip of a cigarette is more harmful than filtered smoke [27]. Our study also found similar results, and our results showed that the RR of passive smoking at home on angina (RR, 1.016) was higher than that of direct smoking (RR, 1.007).

Nevertheless, this study has a few limitations. First, this study merely analyzed associations and not the causal relationships between smoking indices and CVD. It is difficult to consider the temporal relations of smoking indices and CVD with KCHS data because this was a cross-sectional study [18]. In addition, this study did not consider risk factors at the individual level, so the results should be interpreted as a regional association and not as causal relations. Second, we were able to predict regions that were vulnerable to smoking and passive smoking but could not identify the biological and epidemiological causes of their vulnerability. More specifically, this study was able to verify that people with CVD in urban regions (including industrial regions) are relatively more vulnerable to smoking and passive smoking, but we could not shed light on the fundamental hypothesis and cause after adjusting for gender and age. We expect follow-up studies to evaluate the cause and to explain these results via diverse investigations and advanced analytical methods. Finally, this study did not consider social indicators. CVD may be influenced by social factors, such as income level and environment, but this study could not take these into considerations due to a lack of appropriate data. However, we partially compensated for this by examining potential regional features through performing a longitudinal analysis using a random effect model [28,29].

Despite a few limitations, this study offers several benefits. First, the large sample size of more than 1.5 million subjects increased the accuracy of our analysis in identifying the associations between CVD and smoking indices. Particularly, most of the 4 CVD studied were strongly associated with the smoking indices, with stroke having the greatest association with smoking and passive smoking (RR, 1.013 to 1.060). Second, by using an advanced statistical method, we were able to predict RRs not only at the national but also regional levels. The INLA method [20] predicts post-distributions as does the Markov Chain Monte Carlo method that is widely used in Bayesian statistical analysis, but the INLA is quicker and is easier for expanding distributions [30]. This study is valuable in that it simultaneously analyzed national and regional level risks by performing a Bayesian hierarchical analysis, considering regional correlations with the INLA.

Furthermore, the findings of this study can be used as the grounds for public healthcare policies that limit smoking and passive smoking nationwide to promote people’s health. Particularly, they will contribute to devising region-specific healthcare policies for regions populated by groups of people who may be vulnerable to CVD. In other words, there is a need to implement more active measures to limit smoking and to respond to the problems of indoor passive smoking in regions with high populations of people vulnerable to CVD, such as the elderly and financially unstable groups.

## Figures and Tables

**Table 1.** Descriptive statistics for regional prevalence of CVD and confounders

	Diseases	Period	Mean (SD)	Min	25%	Median	75%	Max
CVD (yr)	Hypertension (> 30)	2008-2013	18.2 (2.4)	10.0	16.5	18.3	19.9	25.6
	Stroke (> 50)		3.3 (1.1)	0.4	2.6	3.2	4.0	7.7
	Myocardial infarction (> 40)		1.4 (0.6)	0.1	1.0	1.4	1.8	4.6
	Angina (>40)		1.9 (0.7)	0.2	1.4	1.8	2.3	4.4
Confounders (%)	Smoking rate	2008-2013	25.2 (2.9)	14.9	23.3	25.2	27.2	33.4
	Passive smoking (at home) rate	2009-2011, 2013	11.8 (4.1)	2.3	9.1	11.3	14.1	37.3
	Passive smoking (at work) rate	2009-2011, 2013	29.2 (9.3)	6.0	23.8	28.6	34.9	76.7
	Drinking rate per month	2008-2013	56.5 (5.4)	32.9	53.2	57.1	60.4	68.8
	Body mass index	2008-2013	23.0 (0.7)	22.2	22.8	23.0	23.1	34.5

CVD, cardiovascular disease; SD, standard deviation; Min, minimum; Max, maximum.

**Table 2.** Higher and lower 5 community health centers (si/gun/gu) of cardiovascular disease prevalence

		Si/do	Si/gun/gu	Mean	Standard deviation
Prevalence (%)					
Hypertension (>30 yr)	Higher 5 communities	Gangwon	Sokcho	22.35	1.74
		Incheon	Dong	22.10	2.21
		Gangwon	Goseong	21.98	0.63
		Gangwon	Samcheok	21.88	1.05
		Gangwon	Yeongwol	21.72	2.13
	Lower 5 communities	Gyeongnam	Hapcheon	14.03	1.50
		Jeonbuk	Imsil	14.05	2.85
		Gyeongbuk	Seongju	14.48	1.78
		Jeonnam	Naju	14.50	1.13
		Gyeongnam	Sancheong	14.72	1.15
Stroke (> 50 yr)	Higher 5 communities	Gyeongbuk	Gumi-Gumi	5.33	1.71
		Chungnam	Asan	4.88	1.36
		Gangwon	Yeongwol	4.75	1.79
		Gyeongbuk	Gimcheon	4.72	0.81
		Gyeongbuk	Andong	4.68	0.41
	Lower 5 community health centers	Busan	Suyeong	1.87	0.97
		Jeonnam	Jangheung	1.93	0.75
		Jeonnam	Yeongam	1.97	0.76
		Jeonnam	Wando	2.00	0.58
		Jeonnam	Gangjin	2.12	0.38
Myocardial infarction (> 40 yr)	Higher 5 community health centers	Jeonnam	Damyang	2.38	1.24
		Jeju	Jeju-Jeju	2.28	0.50
		Gyunggi	Goyang-Ilsanseo	2.25	1.18
		Seoul	Nowon	2.20	0.84
		Seoul	Dongjak	2.12	0.44
	Lower 5 community health centers	Gyeongnam	Hamyang	0.68	0.27
		Daegu	Dalseong	0.72	0.40
		Gyeongbuk	Sungju	0.72	0.26
		Jeonbuk	Sunchang	0.78	0.12
		Jeonbuk	Jangsu	0.83	0.32
Angina (> 40 yr)	Higher 5 community health centers	Busan	Yeonje	3.25	0.49
		Gwangju	Nam	2.97	0.69
		Chungbuk	Cheonan	2.92	0.80
		Busan	Dong	2.90	0.18
		Busan	Gijang	2.88	0.96
	Lower 5 community health centers	Gyeongbuk	Sungju	0.87	0.12
		Jeonbuk	Jinan	1.03	0.36
		Ulsan	Dong	1.07	0.34
		Jeonbuk	Wanju	1.08	0.58
		Chungbuk	Jecheon	1.20	0.70
Smoking index (%)					
Smoking rate	Higher 5 community health centers	Gangwon	Taebaek	31.62	1.61
		Gyeonggi	Dongducheon	29.72	1.77
		Chungbuk	Eumseong	29.67	2.80
		Busan	Jung	29.37	2.19
		Gyeonggi	Bucheon -Ojeong	29.35	1.19
	Lower 5 community health centers	Gyeonggi	Gwacheon	17.37	1.68
		Gyeonggi	Seongnam-Bundang	17.50	2.01
		Seoul	Seocho	19.57	1.24
		Gyeonggi	Yongin-Suji	20.00	1.70
		Chungnam	Gyeryong	20.77	1.58
Passive smoking (at home) rate	Higher 5 community health centers	Incheon	Ongjin	24.45	8.65
		Jeju	Dongbu	21.80	3.56
		Jeju	Seogwipo (west)	19.90	6.24
		Jeju	Jeju-Seo(west)	19.88	3.74
		Jeonbuk	Gimje	19.10	8.13
	Lower 5 community health centers	Busan	Suyeong	6.28	2.47
		Gyeongnam	Yangsan	6.28	0.75
		Jeonnam	Gurye	6.73	3.40
		Gyeonggi	Gwacheon	6.78	2.44
		Gangwon	Wonju	6.78	2.42
Passive smoking (at work) rate	Higher 5 community health centers	Chungnam	Dangjin	42.83	24.56
		Ulsan	Nam	41.70	10.22
		Ulsan	Jung	41.40	9.40
		Ulsan	Ulju	40.93	12.45
		Gyeongnam	Changwon-Masan	40.83	5.25
	Lower 5 community health centers	Gyeongbuk	Uiseong	15.70	7.03
		Jeonnam	Gurye	15.83	8.81
		Jeonnam	Goheung	15.85	13.30
		Jeonbuk	Jangsu	17.58	13.55
		Jeonnam	Hampyeong	18.55	4.71

**Table 3.** Relative risk of smoking/passive smoking (at home)/passive smoking (at work) by cardiovascular diseases (per 5% smoking or passive smoking rate)

	Smoking	Exposure to passive smoking	
		Home	Work
Hypertension (> 30 yr)	1.016 (1.004, 1.029)	1.010 (1.006, 1.014)	1.004 (1.002, 1.006)
Stroke (> 50 yr)	1.060 (1.022, 1.100)	1.037 (1.023, 1.051)	1.013 (1.007, 1.019)
Myocardial infarction (> 40 yr)	1.004 (0.958, 1.051)	1.003 (0.986, 1.021)	0.998 (0.991, 1.006)
Angina (> 40 yr)	1.007 (0.966, 1.049)	1.016 (1.001, 1.032)	1.006 (1.000, 1.013)

Values are presented relative risk (95% credibility interval).

**Table 4.** Higher and lower 5 community health centers (si/gun/gu) of relative risk (RR) to smoking/passive smoking (at home)/passive smoking (at work)

		Smoking			Exposure passive smoking					
					Home			Work		
		Si/do	Si/gun/gu	RR	Si/do	Si/gu	RR	Si/do	Si/gu	RR
Hypertension (>30yr)	Higher 5 community health centers	Gyeonggi	Gapyeong	1.046	Gyeonggi	Gapyeong	1.036	Gangwon	Samcheok	1.030
		Gangwon	Samcheok	1.045	Incheon	Dong	1.036	Gyeonggi	Gapyeong	1.030
		Incheon	Dong	1.045	Gangwon	Samcheok	1.035	Incheon	Dong	1.030
		Gangwon	Sokcho	1.042	Gangwon	Sokcho	1.033	Chungcheongnam	Yeongi	1.027
		Gyeonggi	Paju	1.042	Chungnam	Yeongi	1.032	Gangwon	Sokcho	1.027
	Lower 5 community health centers	Gyeongnam	Hapcheon	0.985	Gyeongnam	Hapcheon	0.977	Gyeongnam	Hapcheon	0.972
		Busan	Gangseou	0.991	Busan	Gangseou	0.984	Busan	Gangseou	0.979
		Jeonbuk	Imsil	0.994	Jeonbuk	Imsil	0.987	Jeonbuk	Imsil	0.982
		Jeonbuk	Gochang	0.996	Jeonbuk	Gochang	0.988	Jeonbuk	Gochang	0.984
		Gyeongbuk	Seongju	0.996	Jeju	Seogwipo (west)	0.989	Gyeongbuk	Seongju	0.985
Stroke (>50yr)	Higher 5 community health centers	Gyeongbuk	Gumi-Gumi	1.099	Gyeongbuk	Gumi-Gumi	1.068	Gyeongbuk	Gumi-gumi	1.040
		Daejeon	daedeok	1.099	Gyeonggi	Gapyeong	1.066	Gyeonggi	Gapyeong	1.040
		Gyeonggi	Gapyeong	1.098	Daejeon	Daedeok	1.066	Daejeon	daedeok	1.039
		Gyeongbuk	Cheongdo	1.095	Daegu	Dalseong	1.064	Gyeongbuk	Cheongdo	1.038
		Jeonnam	Asan	1.095	Jeonnam	Asan	1.063	Jeonnam	Asan	1.037
	Higher 5 community health centers	Gyeongnam	Hadong	1.029	Gyeongnam	Hadong	1.005	Gyeongnam	Hadong	0.986
		Chungnam	Wando	1.032	Incheon	Ongjin	1.010	Ulsan	Buk	0.990
		Busan	Suyeong	1.033	Chungnam	Wando	1.011	Busan	Suyeong	0.991
		Ulsan	Dong	1.034	Ulsan	Buk	1.012	Chungnam	Wando	0.991
		Ulsan	Buk	1.034	Busan	Suyeong	1.013	Ulsan	Dong	0.992
Myocardial infarction (> 40 yr)	Higher 5 community health centers	Jeonnam	Yeosu	1.040	Jeonnam	Yeosu	1.039	Jeonnam	Yeosu	1.035
		Daejeon	Dong	1.029	Daejeon	Dong	1.029	Daejeon	Dong	1.024
		Daejeon	Daedeok	1.029	Daejeon	Daedeok	1.028	Daejeon	Daedeok	1.024
		Chungnam	Gongju	1.027	Chungnam	Gongju	1.026	Chungnam	Gongju	1.022
		Jeju	Jeju-Jeju	1.026	Jeju	Jeju-Jeju	1.025	Jeonnam	Gokseong	1.021
	Lower 5 community health centers	Gyeongbuk	Seongju	0.973	Gyeongbuk	Seongju	0.972	Gyeongbuk	Seongju	0.967
		Daegu	Dalseong	0.974	Daegu	Dalseong	0.973	Daegu	Dalseong	0.969
		Gyeongnam	Hamyang	0.977	Gyeongnam	Hamyang	0.977	Gyeongnam	Hamyang	0.972
		Gyeonggi	Sungnam-Sujung	0.980	Gyeonggi	Sungnam-Sujeong	0.980	Gyeonggi	Sungnam-Sujeong	0.975
		Chungnam	Yesan	0.983	Busan	Gangseo	0.983	Chungnam	Yesan	0.978
Angina (> 40 yr)	Higher 5 community health centers	Chungnam	Cheonan	1.047	Chungnam	Cheonan	1.053	Chungnam	Cheonan	1.042
		Jeonbuk	Gimje	1.041	Jeonbuk	Gimje	1.047	Jeonbuk	Gimje	1.038
		Gyeonggi	Gapyeong	1.040	Gyeonggi	Gapyeong	1.047	Gyeonggi	Gapyeong	1.037
		Busan	Gijang	1.038	Busan	Gijang	1.045	Busan	Gijang	1.034
		Gyeonggi	Ansung	1.035	Gyeonggi	Ansung	1.042	Gyeonggi	Ansung	1.032
	Lower 5 community health centers	Gyeongbuk	Seongju	0.975	Gyeongbuk	Seongju	0.985	Gyeongbuk	Seongju	0.976
		Jeonbuk	Wanju	0.985	Jeju	Jeju (west)	0.994	Jeonbuk	Wanju	0.985
		Gyeonggi	Sungnam-Sujung	0.986	Jeonbuk	Wanju	0.995	Gyeonggi	Sungnam-Sujung	0.986
		Jeju	Jeju (west)	0.986	Gyeonggi	Sungnam-Sujung	0.996	Jeju	Jeju (west)	0.986
		Ulsan	Dong	0.989	Incheon	Ganghwa	0.999	Incheon	Ganghwa	0.989

## Appendix 1. Statistical analysis formulation

**Gamma distribution**

$$f(y_{i})=\frac{1}{\Gamma (v)}(\frac{vy}{\mu }{)}^{v}exp(\frac{-vy}{\mu }),y\geq 0,\mu >0,v>0\;Y_{i}\sim \Gamma (\mu ,v)\;\log(v)\sim \mathrm{logGamma}(1,\;0.01)$$

where *Y_i_* is a observed values with *i*= 1, 2, … N. We assumed Gamma distribution as a distribution of *Y_i_* with parameter *μ* and *ν*. And we set the prior distribution of log(*ν*) as logGamma distribution with parameter 1, and 0.01 each.

**Integrated nested Laplace approximation (INLA)**

p(yβ, ψ) = ∏i=1np(yiβi, ψ)p(βψ)=(2π)-n2Q(ψ)12exp(-12β'Q(ψ)β)

The INLA algorithm, proposed by Rue al. (2009) [1] and Martino & Rue [2] is a deterministic algorithm for Bayesian inference rather than simulation based, MCMC (Markov Chain Monte Carlo). β is a set of regression 16 coefficient parameter and Q(ψ) is a sparse precision matrix known as Gaussian Markov random field (GMRF).

**Regression model**

$$\log(\mu_{ij})\sim (\beta_{0}+b_{0j}+b_{2j})+(\beta_{1}+b_{1j})X_{ij}+\beta_{-0,1}Z\;b_{0j}\sim Normal(0,\sigma_{b0}^{2}),b_{1j}\sim Normal(0,\sigma_{b1}^{2})\;1/\sigma_{v0}^{2}\sim \log Gamma(1,0.00005),1/\sigma_{v1}^{2}\sim \log Gamma(1,0.00005)$$

*β*_0_ is an intercept of regression model and *X* is an interesting variable (smoking, indirect smoking indices, each). Bayesian hierarchical model is used with hyper parameters;ψ=($\sigma_{v0}^{2}$, $\sigma_{v1}^{2}$ etc).

**Besag-York-Mollie (BYM) model**

b2j=uj+vjuju-j~Normal(1Ni∑ kajkuk,sj2),vj=Normal(0,σv2)

Considering N areas, each characterized by a set of neighbors *N_j_*. And *a_jk_* is 1 if areas j and k are neighbors and 0 otherwise.

$s_{j}^{2}=\sigma_{u}^{2}/N_{j}$ is the variance for the same area. *v_j_* is the area-specific effect modeled as exchangeable.
