# Supplementary material for: The association between smoking or passive smoking and cardiovascular diseases using a Bayesian hierarchical model: based on the 2008-2013 Korea Community Health Survey
Source: Epidemiol Health. 2017 Jun 22;39:e2017026. doi: 10.4178/epih.e2017026 (PMC5723911; doi:10.4178/epih.e2017026)
Supplement: Supplementary file 1 [file epih-39-e2017026-supplementary.pdf]

Open Access

Volume: 39, Article ID: e2017026, 10 pages
https://doi.org/10.4178/epih.e2017026

epiH Epidemiology and Health

ORIGINAL ARTICLE

베이지안 계층화 방법을 이용한 흡연 및 간접 흡연과 심혈관계 질환 유병률의 상관성 분석: 2008-2013 지역사회 건강조사 자료를 이용하여

이환희, 황성희, 최하영, 김호

서울대학교 보건대학원

The association between smoking or passive smoking and cardiovascular diseases using a Bayesian hierarchical model: based on the 2008-2013 Korea Community Health Survey

Whanhee Lee, Sung-Hee Hwang, Hayoung Choi, Ho Kim

Graduate School of Public Health, Seoul National University, Seoul, Korea

KOREAN SUMMARY

베이지안 계층화 방법을 이용하여 흡연 및 간접 흡연과 심혈관계 질환 유병의 전국 및 시/군/구별 연관성을 추정한 결과, 흡연은 뇌졸중(RR=1.060)과 고혈압(RR=1.016) 유병에 유의하게 연관되었다.

Objective: Smoking and passive smoking have been extensively reported as risk factors of cardiovascular morbidity and mortality. Despite the biological mechanisms underlying the impact of hazardous chemical substances contained in tobacco in cardiovascular diseases (CVD), studies investigating the association between smoking and passive smoking with morbidity are at an inchoate stage in Korea. Therefore, this study aimed to estimate the risks of smoking and passive smoking on cardiovascular morbidity at the national and regional levels.

Methods: This study calculated sex-standardized and age-standardized prevalence of CVD and smoking indices in 253 community health centers (si/gun/gu) in Korea using the 2008-2013 Korea Community Health Survey data. Furthermore, a Bayesian hierarchical model was used to estimate the association of smoking and passive smoking with the prevalence of CVD from the national and regional community health centers.

Results: At the national level, smoking was significantly associated with stroke (relative risk [RR], 1.060) and hypertension (RR, 1.016) prevalence, whilst passive smoking at home and work were also significantly associated with prevalence of stroke (RR, 1.037/1.013), angina (RR, 1.016/1.006), and hypertension (RR, 1.010/1.004). Furthermore, the effects of smoking and passive smoking were greater in urban-industrial areas than in rural areas.

Conclusion: The findings of this study would provide grounds for national policies that limit smoking and passive smoking, as well as regionally serve as the basis for region-specific healthcare policies in populations with high CV D vulnerability.]

KEY WORDS: Smoking, Passive smoking, Cardiovascular diseases, Korea Community Health Survey, Korea

Correspondence: Ho Kim
Graduated School of Public Health, Seoul National University, 1 Gwanak-ro, Gwanak-gu, Seoul 08826, Korea
E-mail: hokim@snu.ac.kr
Received: Jan 2, 2017 / Accepted: Jan 17, 2017 / Published: Jun 22, 2017

This article is available from: http://e-epih.org/

ⓒ This is an open-access article distributed under the terms of the Creative Commons Attribution License (http://creativecommons.org/licenses/by/4.0/), which permits unrestricted use, distribution, and reproduction in any medium, provided the original work is properly cited.

ⓒ 2017, Korean Society of Epidemiology

서 론

기존의 수 많은 연구를 통해 흡연 및 간접흡연이 여러 질병의 발생 및 사망의 위험인자라는 것은 잘 알려져 있다[1-4]. 특히 담배가 가지고 있는 4,000여 종류 이상의 화학 물질 중 다환방향족 탄화수소(PAHs: polycyclic aromatic hydrocarbons)와 산화기체는 심장독성을 일으켜[1], 관상동맥질환, 허혈성 뇌졸중 및 비외상성 자주막하 출혈 등 심혈관계 질환에 부정적인 영향을 미친다고 보고되었다[5, 6].

세계 보건기구 연구 결과 흡연으로 인한 사망 중 심혈관계 질환 관련 사망(169만명)이 가장 빈번한 것으로 조사되었으며, 미국 남성 36만명을 대상으로 한 연구에서 흡연은 폐암, 만성심장질환 및 뇌졸중의 주요 위험 인자라고 보고되었다[3]. 노르웨이의 연구에서도 흡연자가 비흡연자에 비해 뇌졸중 발병은 2.74배, 그로 인한 사망은 6.74배 높은 것으로 나타났다[4]. 또한 흡연과 유사하게, 간접흡연 또한 비흡연 성인의 호흡기 및 심혈관 질환, 각종 암, 조기 사망의 원인이 된다고 보고되었다[7]. 이외에 다른 연구들에서는 비흡연자가 간접흡연에 노출되었을 경우 그렇지 않았을 때보다 심장질환 위험을 25-30% 증가시키는 것으로 나타났다[8-10].

한국에서도 흡연이 성인의 심혈관 질환 관련 사망 위험을 증가시킨다는 연구가 있었다[11-13]. 국내 흡연 관련하여 사망한 질병별 사망자수는 2012년 기준 4,148명(뇌졸중), 3,858명(허혈성 심장질환)으로 보고되었으며, 이는 전체 질병 중 두 번째, 세 번째로 높은 순위를 차지하였다[14]. 또한 한국에서 성인 남녀를 대상으로 발생한 심혈관계질환 관련 사망 중 26.7%가 흡연에 기인하였다고 보고되기도 하였다[13]. 한국의 성인 흡연율은 1998년 이후 꾸준히 감소하는 추세에 있지만[15], 과거 흡연이 사망에 영향을 미칠 수 있으며[11] 고혈압 및 심장질환, 대뇌혈관질환으로 인한 진료비 및 진료인원 또한 꾸준하고 증가하는 추세이기 때문에[16], 흡연은 앞으로도 심혈관계 질환의 유병과 사망에 있어 지속적으로 관심을 가져야 할 건강유해요인임이 분명하다.

이렇듯 국내에서 흡연과 심혈관계 질환 사망의 연관성 연구는 많이 진행되었지만, 흡연과 유병에 관한 연구는 상대적으로 적은 편이다. 그러므로 본 연구에서는 흡연과 심혈관계 질환(고혈압, 뇌졸중, 심근경색, 및 협심증) 유병에 대한 연관성을 정량적으로 분석하고, 흡연이 심혈관계 질환 유병에 미치는 상대 위험도를 150만명 이상이 참여한 대규모 단면 조사 연구(Cross-sectional study) 자료를 이용하여 추정하는 것을 목표로 하였다. 나아가 흡연뿐만 아니라 간접 흡연 또한 지표로 사용하여, 간접 흡연이 심혈관계 질환 유병에 미치는 영향도 추정하고자 하였다.

연구대상 및 방법

연구자료

본 연구에서는 2008년부터 2013년까지 한국의 253개 시/군/구 보건소에서 시행된 지역사회 건강조사 (Korea Community Health Survey, KCHS) 자료를 이용하였다 [17]. KCHS는 한국질병관리본부에서 시행되고 있으며, 전국적으로 19세 이상의 성인들을 대상으로 인터뷰를 진행하여 자료를 수집한다. KCHS 표본 추출은 동/읍/면 내에서 주택유형에 따라 시/군/구당 평균 900명 을 대상으로 하며, 표본추출은 확률비례계통추출법으로 1차 표본지점을 추출 한 후, 계통추출법으로 2차 표본가구를 선정하여 이루어진다 [18]. 본 연구에서 사용된 KCHS 자료는 총 1,567,930명이 포함되었으며 (2008년: 200,800명, 2009년: 220,258명, 2010년: 230,712명, 2011년: 229,229명, 2012년: 229,226명, 2013년: 228,781명), 매년 임의로 추출되었다. 이 자료를 이용하여 연구 연도의 인구에 기준한 성-연령이 고려된 시/군/구별 4개 심혈관계질환 (30세 이상 고혈압, 50세 이상 뇌졸중, 40세 이상 심근경색, 및 40세 이상 협심증) 유병률과 흡연관련 지역 지표 (현재 흡연율, 가정 내 간접흡연 비율, 및 직장 내 간접흡연 비율), 평균 체질량지수 (BMI), 월간음주율을 계산하였다. 심혈관계질환 유병률은 자가 기록된 평생 의사 진단 여부를 기준으로 계산되었으며, BMI, 월간음주율 또한 자가 기입된 자료를 바탕으로 하였다.

Table 1. Descriptive statistics for regional prevalence of CVD and confounders

	Diseases	Period	Mean (SD)	Min	25%	Median	75%	Max
CVD (yr)	Hypertension (> 30)	2008-2013	18.2 (2.4)	10.0	16.5	18.3	19.9	25.6
	Stroke (> 50)		3.3 (1.1)	0.4	2.6	3.2	4.0	7.7
	Myocardial infarction (> 40)		1.4 (0.6)	0.1	1.0	1.4	1.8	4.6
	Angina (>40)		1.9 (0.7)	0.2	1.4	1.8	2.3	4.4
Confounders (%)	Smoking rate	2008-2013	25.2 (2.9)	14.9	23.3	25.2	27.2	33.4
	Passive smoking (at home) rate	2009-2011, 2013	11.8 (4.1)	2.3	9.1	11.3	14.1	37.3
	Passive smoking (at work) rate	2009-2011, 2013	29.2 (9.3)	6.0	23.8	28.6	34.9	76.7
	Drinking rate per month	2008-2013	56.5 (5.4)	32.9	53.2	57.1	60.4	68.8
	Body mass index	2008-2013	23.0 (0.7)	22.2	22.8	23.0	23.1	34.5

SD, standard deviation; CVD, cardiovascular disease; Min, minimum; Max, maximum.

Table 2. Higher and lower 5 community health centers (si/gun/gu) of cardiovascular disease prevalence

		Si/do	Si/gun/gu	Mean	Standard deviation
Prevalence (%)					
Hypertension (>30 yr)	Higher 5 communities	Gangwon	Sokcho	22.35	1.74
		Incheon	Dong	22.10	2.21
		Gangwon	Goseong	21.98	0.63
		Gangwon	Samcheok	21.88	1.05
		Gangwon	Yeongwol	21.72	2.13
	Lower 5 communities	Gyeongnam	Hapcheon	14.03	1.50
		Jeonbuk	Imsil	14.05	2.85
		Gyeongbuk	Seongju	14.48	1.78
		Jeonnam	Naju	14.50	1.13
		Gyeongnam	Sancheong	14.72	1.15
Stroke (> 50 yr)	Higher 5 communities	Gyeongbuk	Gumi-Gumi	5.33	1.71
		Chungnam	Asan	4.88	1.36
		Gangwon	Yeongwol	4.75	1.79
		Gyeongbuk	Gimcheon	4.72	0.81
		Gyeongbuk	Andong	4.68	0.41
	Lower 5 community health centers	Busan	Suyeong	1.87	0.97
		Jeonnam	Jangheung	1.93	0.75
		Jeonnam	Yeongam	1.97	0.76
		Jeonnam	Wando	2.00	0.58
		Jeonnam	Gangjin	2.12	0.38
Myocardial infarction (> 40 yr)	Higher 5 community health centers	Jeonnam	Damyang	2.38	1.24
		Jeju	Jeju-Jeju	2.28	0.50
		Gyunggi	Goyang-Ilsanseo	2.25	1.18
		Seoul	Nowon	2.20	0.84
		Seoul	Dongjak	2.12	0.44
	Lower 5 community health centers	Gyeongnam	Hamyang	0.68	0.27
		Daegu	Dalseong	0.72	0.40
		Gyeongbuk	Sungju	0.72	0.26
		Jeonbuk	Sunchang	0.78	0.12
		Jeonbuk	Jangsu	0.83	0.32
Angina (> 40 yr)	Higher 5 community health centers	Busan	Yeonje	3.25	0.49
		Gwangju	Nam	2.97	0.69
		Chungbuk	Cheonan	2.92	0.80
		Busan	Dong	2.90	0.18
		Busan	Gijang	2.88	0.96
	Lower 5 community health centers	Gyeongbuk	Sungju	0.87	0.12
		Jeonbuk	Jinan	1.03	0.36
		Ulsan	Dong	1.07	0.34
		Jeonbuk	Wanju	1.08	0.58
		Chungbuk	Jecheon	1.20	0.70
Smoking index (%)					
Smoking rate	Higher 5 community health centers	Gangwon	Taebaek	31.62	1.61
		Gyeonggi	Dongducheon	29.72	1.77
		Chungbuk	Eumseong	29.67	2.80
		Busan	Jung	29.37	2.19
		Gyeonggi	Bucheon -Ojeong	29.35	1.19
	Lower 5 community health centers	Gyeonggi	Gwacheon	17.37	1.68
		Gyeonggi	Seongnam -Bundang	17.50	2.01
		Seoul	Seocho	19.57	1.24
		Gyeonggi	Yongin -Suji	20.00	1.70
		Chungnam	Gyeryong	20.77	1.58

(Continued to the next page)

Table 2. Continued

		Si/do	Si/gun/gu	Mean	Standard deviation
Passive smoking (at home) rate	Higher 5 community health centers	Incheon	Ongjin	24.45	8.65
		Jeju	Dongbu	21.80	3.56
		Jeju	Seogwipo (west)	19.90	6.24
		Jeju	Jeju -Seo(west)	19.88	3.74
		Jeonbuk	Gimje	19.10	8.13
	Lower 5 community health centers	Busan	Suyeong	6.28	2.47
		Gyeongnam	Yangsan	6.28	0.75
		Jeonnam	Gurye	6.73	3.40
		Gyeonggi	Gwacheon	6.78	2.44
		Gangwon	Wonju	6.78	2.42
Passive smoking (at work) rate	Higher 5 community health centers	Chungnam	Dangjin	42.83	24.56
		Ulsan	Nam	41.70	10.22
		Ulsan	Jung	41.40	9.40
		Ulsan	Ulju	40.93	12.45
		Gyeongnam	Changwon -Masan	40.83	5.25
	Lower 5 community health centers	Gyeongbuk	Uiseong	15.70	7.03
		Jeonnam	Gurye	15.83	8.81
		Jeonnam	Goheung	15.85	13.30
		Jeonbuk	Jangsu	17.58	13.55
		Jeonnam	Hampyeong	18.55	4.71

Table 3. Relative risk of smoking/passive smoking (at home)/passive smoking (at work) by cardiovascular diseases (per 5% smoking or passive smoking rate)

	Smoking	Exposure to passive smoking	
		Home	Work
Hypertension (> 30 yr)	1.016 (1.004, 1.029)	1.010 (1.006, 1.014)	1.004 (1.002, 1.006)
Stroke (> 50 yr)	1.060 (1.022, 1.100)	1.037 (1.023, 1.051)	1.013 (1.007, 1.019)
Myocardial infarction (> 40 yr)	1.004 (0.958, 1.051)	1.003 (0.986, 1.021)	0.998 (0.991, 1.006)
Angina (> 40 yr)	1.007 (0.966, 1.049)	1.016 (1.001, 1.032)	1.006 (1.000, 1.013)

Values are presented relative risk (95% credibility interval).

통계분석

2008년부터 2013년까지 반복적으로 측정된 흡연 지표와 심혈관계질환 유병률의 연관성을 전국적/지역적으로 분석하기 위해서, 일반화 혼합효과 모형(Generalized Linear Mixed Model)이 사용되었다. 반응변수로는 네 가지 심혈관계 질환(30세 이상 고혈압, 50세 이상 뇌졸중, 40세 이상 심근경색, 및 40세 이상 협심증)의 유병률이 사용되었다. 이 네 유병률에 대하여 정규성 검정(Shapiro-wilk Test)를 수행한 결과, 정규분포를 따르지 않는 것으로 확인하였으며, 감마분포의 형태를 띄는 것을 확인하여 반응변수를 감마분포로 가정한 일반화 혼합모형을 사용하였다. 연결(Link) 함수로는 Log 함수가 사용되었다. 교란변수 및 공변량으로는 2008-2013 시/군/구 평균 체질량지수(BMI)와 월간음주율을 사용하였으며, 시간의 효과를 고려하기 위해 선형으로 연간 효과를 보정하였다. 흡연 지표들(현재 흡연율, 가정내 간접흡연율, 직장 내 간접흡연율)은 반응변수로 사용되었으며, 교란변수 및 공변량이 동일한 상태에서 반응변수와의 연관성을 각각 추정하였다.

또한 시/군/구별 흡연 지표의 영향을 측정하고, 거리에 따른 상관성을 보정하기 위해서 베이지안 계층화 모형(Bayesian Hierarchical Model)을 일반화 혼합효과 모형과 함께 사용하였다. 시/군/구별 흡연 지표와 심혈관계 질환의 연관성은 임의 절편(Random Intercept)와 임의 기울기(Random Slope)을 이용하였으며, 임의절편과 임의 기울기의 사전(Prior)분포는 각각 평균이 0인 정규분포를 따르며, 분산의 역수는 감마분포(모수=1, 0.00005)를 따른다고 가정하였다. 공간 상관성은 Besag-York-Mollie (BYM)방법 [19]을 사용하여 임의 절편(Random Intercept)으로 보정하였고, 모든 임의 절편과 임의 기울기는 서로 독립임을 가정하였다. 나아가 기존 연구에 근거하여 임의 효과가 고려된(Conditioned) 고정 효과(Fixed Effect)는 분산을 Gaussian Markov Random Field로 가정

Table 4. Higher and lower 5 community health centers (si/gun/gu) of relative risk (RR) to smoking/passive smoking (at home)/passive smoking (at work)

		Smoking			Exposure passive smoking					
					Home			Work		
		Si/do	Si/gun/gu	RR	Si/do	Si/gu	RR	Si/do	Si/gu	RR
Hyperten-sion (> 30 yr)	Higher 5 community health centers	Gyeonggi	Gapyeong	1.046	Gyeonggi	Gapyeong	1.036	Gangwon	Samcheok	1.030
		Gangwon	Samcheok	1.045	Incheon	Dong	1.036	Gyeonggi	Gapyeong	1.030
		Incheon	Dong	1.045	Gangwon	Samcheok	1.035	Incheon	Dong	1.030
		Gangwon	Sokcho	1.042	Gangwon	Sokcho	1.033	Chungcheongnam	Yeongi	1.027
		Gyeonggi	Paju	1.042	Chungnam	Yeongi	1.032	Gangwon	Sokcho	1.027
	Lower 5 community health centers	Gyeongnam	Hapcheon	0.985	Gyeongnam	Hapcheon	0.977	Gyeongnam	Hapcheon	0.972
		Busan	Gangseou	0.991	Busan	Gangseou	0.984	Busan	Gangseou	0.979
		Jeonbuk	Imsil	0.994	Jeonbuk	Imsil	0.987	Jeonbuk	Imsil	0.982
		Jeonbuk	Gochang	0.996	Jeonbuk	Gochang	0.988	Jeonbuk	Gochang	0.984
		Gyeongbuk	Seongju	0.996	Jeju	Seogwipo (west)	0.989	Gyeongbuk	Seongju	0.985
Stroke (> 50 yr)	Higher 5 community health centers	Gyeongbuk	Gumi-Gumi	1.099	Gyeongbuk	Gumi-Gumi	1.068	Gyeongbuk	Gumi-gumi	1.040
		Daejeon	daedeok	1.099	Gyeonggi	Gapyeong	1.066	Gyeonggi	Gapyeong	1.040
		Gyeonggi	Gapyeong	1.098	Daejeon	Daedeok	1.066	Daejeon	daedeok	1.039
		Gyeongbuk	Cheongdo	1.095	Daegu	Dalseong	1.064	Gyeongbuk	Cheongdo	1.038
		Jeonnam	Asan	1.095	Jeonnam	Asan	1.063	Jeonnam	Asan	1.037
	Lower 5 community health centers	Gyeongnam	Hadong	1.029	Gyeongnam	Hadong	1.005	Gyeongnam	Hadong	0.986
		Chungnam	Wando	1.032	Incheon	Ongjin	1.010	Ulsan	Buk	0.990
		Busan	Suyeong	1.033	Chungnam	Wando	1.011	Busan	Suyeong	0.991
		Ulsan	Dong	1.034	Ulsan	Buk	1.012	Chungnam	Wando	0.991
		Ulsan	Buk	1.034	Busan	Suyeong	1.013	Ulsan	Dong	0.992
Myocardial infarction (> 40 yr)	Higher 5 community health centers	Jeonnam	Yeosu	1.040	Jeonnam	Yeosu	1.039	Jeonnam	Yeosu	1.035
		Daejeon	Dong	1.029	Daejeon	Dong	1.029	Daejeon	Dong	1.024
		Daejeon	Daedeok	1.029	Daejeon	Daedeok	1.028	Daejeon	Daedeok	1.024
		Chungnam	Gongju	1.027	Chungnam	Gongju	1.026	Chungnam	Gongju	1.022
		Jeju	Jeju-Jeju	1.026	Jeju	Jeju-Jeju	1.025	Jeonnam	Gokseong	1.021
	Lower 5 community health centers	Gyeongbuk	Seongju	0.973	Gyeongbuk	Seongju	0.972	Gyeongbuk	Seongju	0.967
		Daegu	Dalseong	0.974	Daegu	Dalseong	0.973	Daegu	Dalseong	0.969
		Gyeongnam	Hamyang	0.977	Gyeongnam	Hamyang	0.977	Gyeongnam	Hamyang	0.972
		Gyeonggi	Sungnam-Sujung	0.980	Gyeonggi	Sungnam-Sujeong	0.980	Gyeonggi	Sungnam-Sujeong	0.975
		Chungnam	Yesan	0.983	Busan	Gangseo	0.983	Chungnam	Yesan	0.978
Angina (> 40 yr)	Higher 5 community health centers	Chungnam	Cheonan	1.047	Chungnam	Cheonan	1.053	Chungnam	Cheonan	1.042
		Jeonbuk	Gimje	1.041	Jeonbuk	Gimje	1.047	Jeonbuk	Gimje	1.038
		Gyeonggi	Gapyeong	1.040	Gyeonggi	Gapyeong	1.047	Gyeonggi	Gapyeong	1.037
		Busan	Gijang	1.038	Busan	Gijang	1.045	Busan	Gijang	1.034
		Gyeonggi	Ansung	1.035	Gyeonggi	Ansung	1.042	Gyeonggi	Ansung	1.032
	Lower 5 community health centers	Gyeongbuk	Seongju	0.975	Gyeongbuk	Seongju	0.985	Gyeongbuk	Seongju	0.976
		Jeonbuk	Wanju	0.985	Jeju	Jeju (west)	0.994	Jeonbuk	Wanju	0.985
		Gyeonggi	Sungnam-Sujung	0.986	Jeonbuk	Wanju	0.995	Gyeonggi	Sungnam-Sujung	0.986
		Jeju	Jeju (west)	0.986	Gyeonggi	Sungnam-Sujung	0.996	Jeju	Jeju (west)	0.986
		Ulsan	Dong	0.989	Incheon	Ganghwa	0.999	Incheon	Ganghwa	0.989

한 다변량 정규분포(Multivariate Normal)를 사전분포로 가정하였으며[20], 앞선 사전분포들을 바탕으로 사후 신용구간(Posterior Credibility Interval; C.I.)를 추정하였다. 모든 분석은 R과 R 패키지(버전 3.2.2) *INLA* (Integrated Nested Laplace Approximation) [21]을 이용하여 수행되었다. 자세한 수식은 Appendix의 Statistical Analysis formulation에 기재되어 있으며, 예측된 사후 분포 평균과 관측값을 그린 그림 또한 Appendix (Appendix 1)에 기재되어 있다.

연구결과

Table 1은 본 연구에 사용된 변수들의 기술통계량이다. 심혈관계 질환 중에서는 30세 이상 고혈압 (Hypertension over 30 years)이 6년 (2008-2013)동안 18.2%로 유병률이 가장 높았으며, 40세 이상 협심증 (Angina over 40 years)의 유병률이 1.9%로 가장 낮았다. 유병률의 분포를 보았을 때, 전국적으로 유병률의 분포가 다양한 것을 확인할 수 있었다. 관심변수로서 흡연율은 6년 평균 25.2% (14.9-33.4%), 로 관찰되었으며, 가정 내 간접 흡연율은 평균 11.8% (2.3-37.3%), 직장 내 간접 흡연율은 평균 29.2% (6-76.7%)로 나타났다. 253개 시/군/구에 대한 기본적인 정보 (인구 및 소득 수준)은 Appendix 2에 기술되어 있다.

Table 2는 6년 평균 심혈관계 질환 유병률과 흡연 지표 상-하위 5개 시/군/구를 보여준다. 30세 이상 고혈압 유병률은 강원도 소재 시/군/구에서 대체적으로 높았으며(21.72-22.35%), 경상도와 전라도 소재 시/군/구에서 낮았다(14.03-14.72%). 50세 이상 뇌졸중 유병률(Stroke over 50 years)은 경상북도 소재 시/군/구에서 높았으며(4.68-5.33%), 전라남도 소재 시/군/구에서 낮았다(1.93-2.12%). 40세 이상 심근경색 유병률(Myocardial Infarction over 40 years)

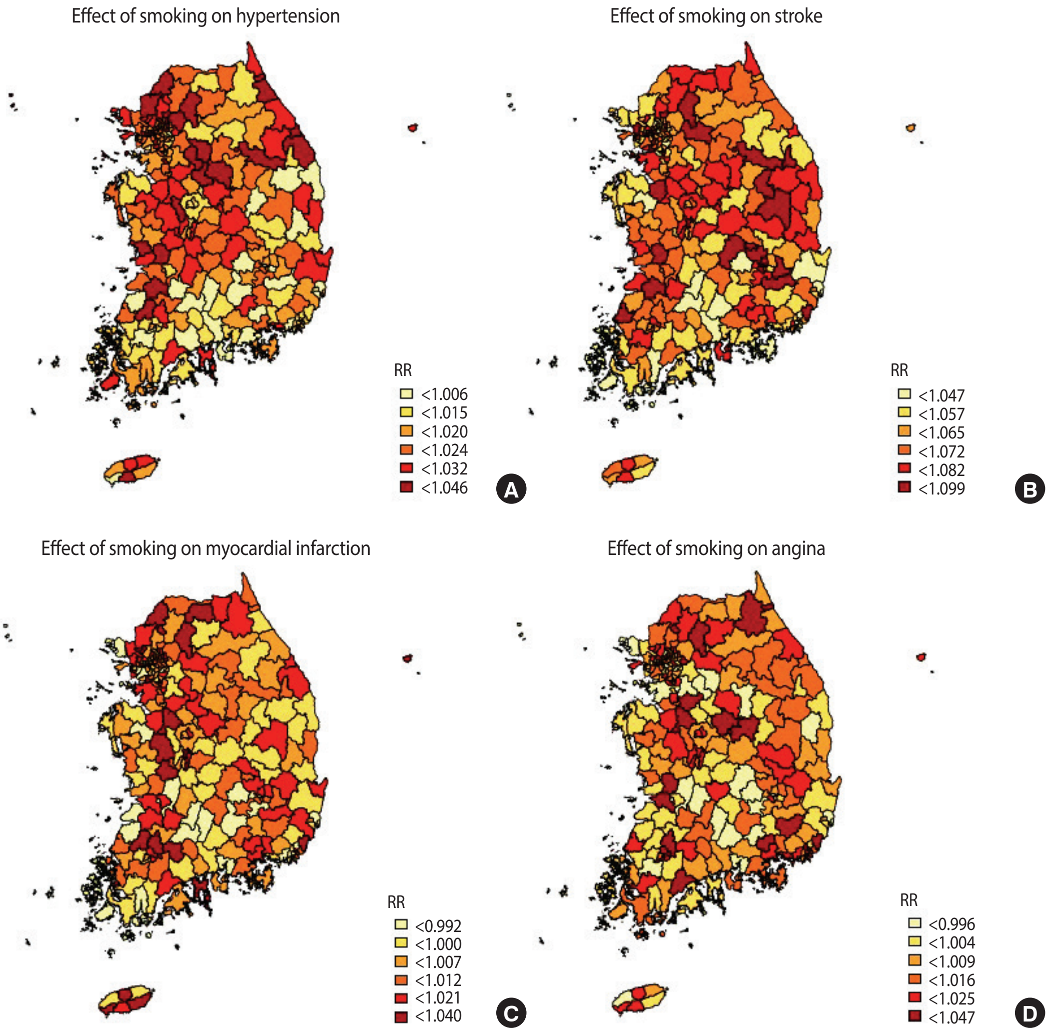

Figure 1. Geographical distribution of relative risks (RR). The associations between 4 cardiovascular diseases (A) hypertension over 30 years, (B) stoke over 50 years, (C) myocardial infarction over 40 years, and (D) angina over 40 years and smoking.

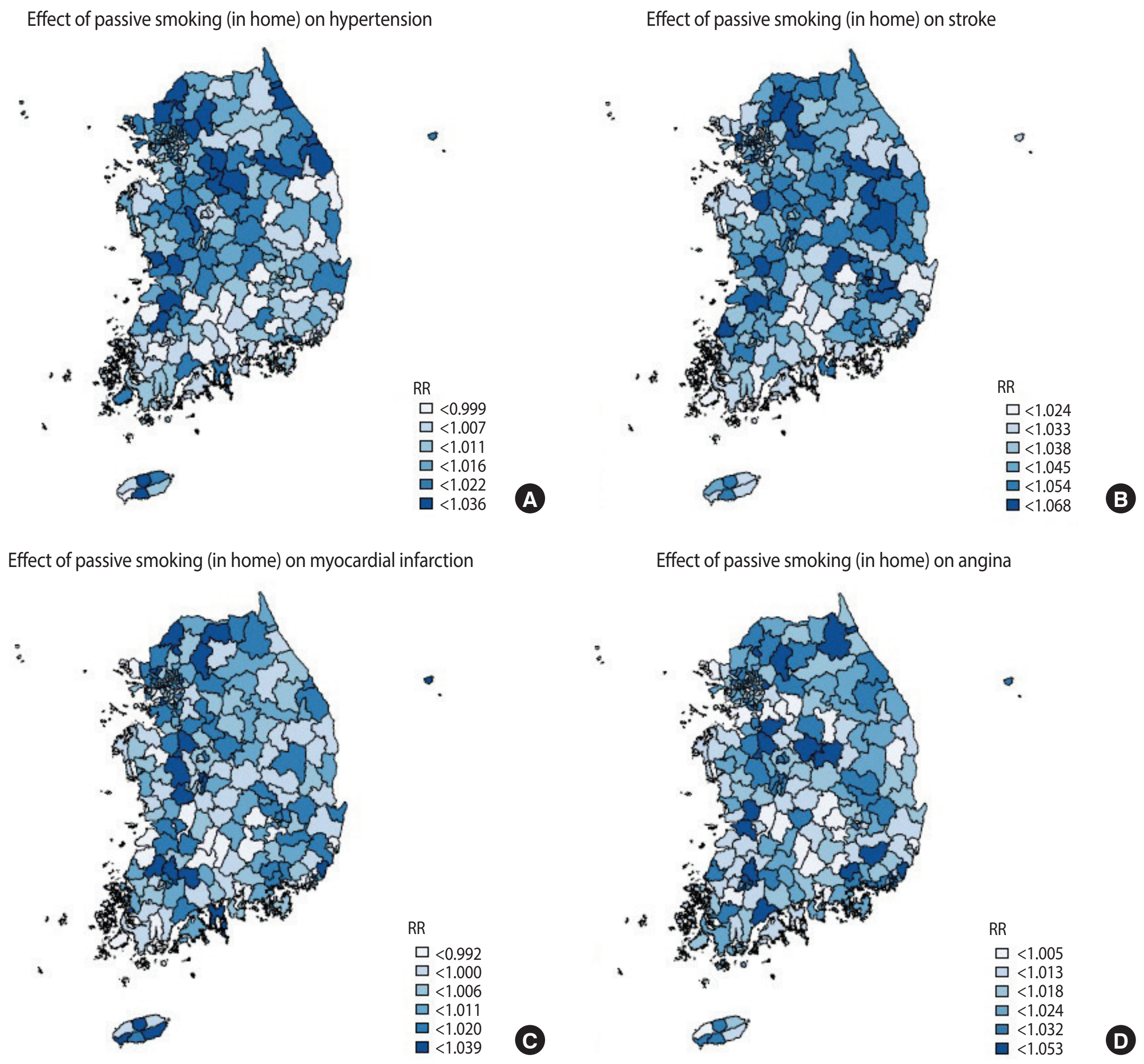

Figure 2. Geographical distribution of relative risks (RR). The associations between 4 cardiovascular diseases (A) hypertension over 30 years, (B) stoke over 50 years, (C) myocardial infarction over 40 years, and (D) angina over 40 years and passive smoking at home.

은 서울특별시와 경기도 소재 시/군/구에서 높은 경향을 보였으며 (2.12-2.25%), 전라북도 소재 시/군/구에서는 낮았다(0.78-0.83%). 40세 이상 협심증 유병률은 부산광역시 시/군/구가 높은 경향을 보였으며(2.88-3.25%), 전북에서 낮았다(1.03-1.08%). 흡연 지표의 경우, 흡연율은 지역적 특성이 보이지 않았으며, 가정 내 간접 흡연율은 제주도 소재 시/군/구가 높았으며(19.88-21.80%), 직장 내 간접 흡연율은 울산광역시 소재 시/군/구에서 높은 특성을 보였다 (40.93-41.70%).

흡연 지표가 심혈관계 질환 유병률에 미치는 전국 평균 상대 위험도(Relative Risk; RR)는 Table 3에 제시되어 있으며, 지역별 (상-하위 5개 지역) 상대 위험도는 Table 4에 기술되어 있다. Table 3-4의 모든 상대 위험도는 흡연 지표 5% 증가시의 상대 위험도이다. Table 3에서 흡연율은 뇌졸중과 가장 큰 연관성(RR=1.060, 95% Credibility Interval (C.I)=1.022-1.100)을 보였으며, 고혈압 또한 유의한 연관성을 보였다(RR=1.016, 95% C.I.=1.004-1.029). 가정 내 간접흡연 또한 뇌졸중 유병률 증가와 가장 큰 연관성을 보였으며(RR=1.037, 95% C.I.=1.023-1.051), 심근경색을 제외하고 다른 세 질환에서 모두 통계적으로 유의한 상대위험도를 보였다(RR=1.010-1.037). 직장 내 간접흡연도 뇌졸중에 미치는 영향이 가장 컸으며(RR=1.013, 95%C.I.=1.007-1.019), 고혈압에 미치는 영향 또한 유의하였다(RR=1.004, 95%C.I.=1.002-1.006).

Table 4에는 흡연 지표의 상대위험도가 가장 높은/낮은 3개 시/군/구가 기술되어 있으며, Figure 1-3에는 시/군/구 단위 흡연율과 가정/직장 내 간접흡연율이 심혈관계 질환 유병률에 미치는 영향이 전국 지도로 표시되어 있다. 흡연율의 고혈압 유병률에 대한 상대 위험도는 경기도와 강원 지역의 시/군/구에서 높게 나타났으며

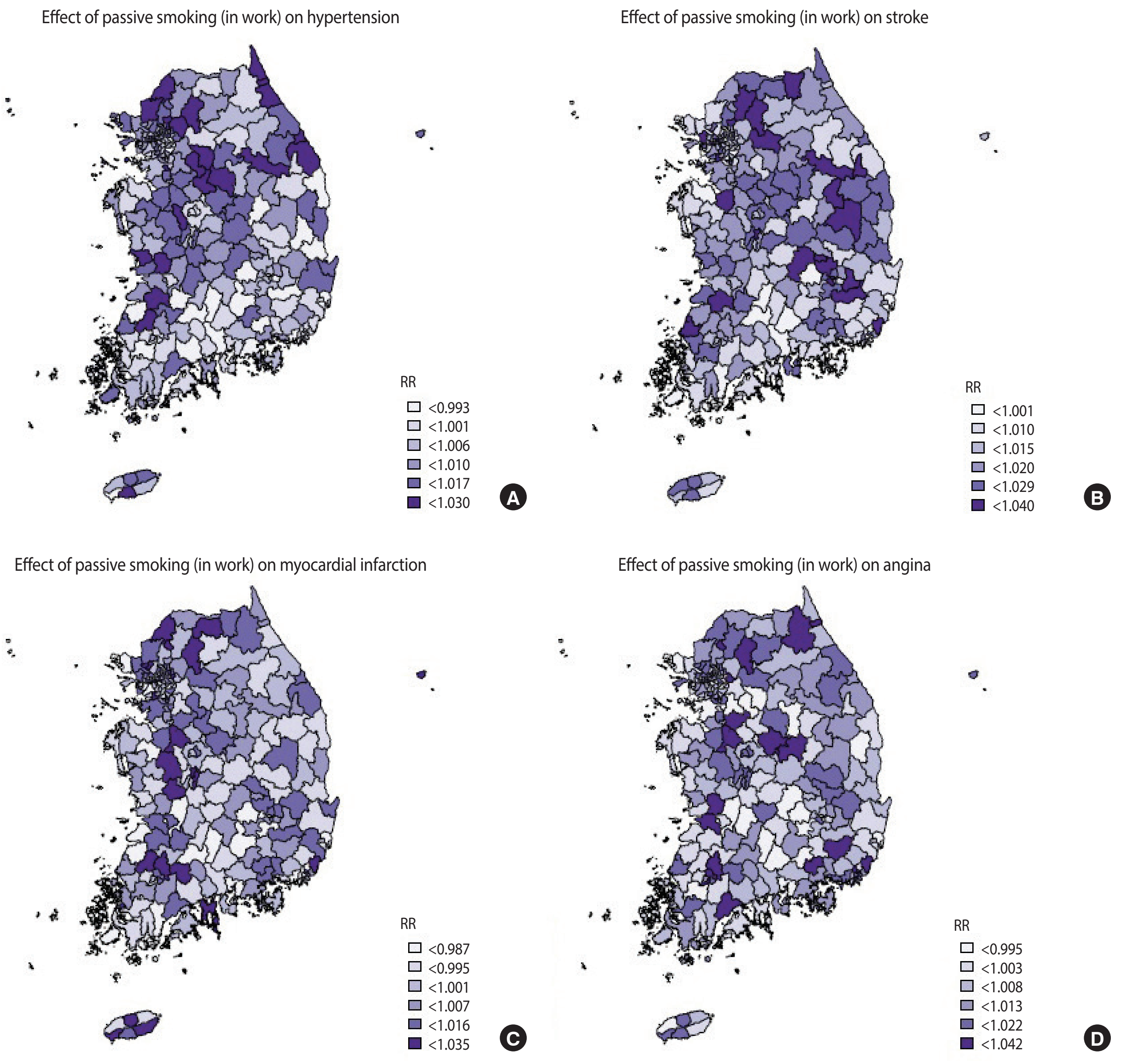

Figure 3. Geographical distribution of relative risks (RR). The associations between 4 cardiovascular diseases (A) hypertension over 30 years, (B) stoke over 50 years, (C) myocardial infarction over 40 years, and (D) angina over 40 years and passive smoking at work.

(RR=1.042-1.046), 전북과 경상도 지역 시/군/구에서는 낮게 나타났다(RR=0.985-0.996). 또한 흡연은 경상북도에서 시/군/구에서 뇌졸중 유병과 연관이 높았으며(RR=1.095-1.099), 부산과 울산 시/군/구에서는 낮았다(RR=1.033-1.034). 심근경색은 대전 시/군/구에서 높았으며(RR=1.029-1.029), 상대 위험도가 낮은 지역(RR=0.973-0.983)은 지역적 특성을 보이지 않았다. 나아가 흡연이 협심증에 미치는 영향은 경기지역에서 높게 나타났고(RR=1.040-1.035), 한반도 남부 지역(경상북도, 전라북도, 제주도 등)에서 낮게 나타났다(RR=0.975-0.989). 가정 및 직장 내 간접흡연의 상대 위험도 분포는 흡연과 비슷한 형태를 띄었으며, 시/군/구 순위 또한 유사하였다. 또한 가정 내 간접흡연 노출(RR=1.003-1.037)은 모든 심혈관계 질환에서 직장 내 간접흡연 노출(RR=0.998-1.013)보다 영향이 컸다. 또한 전체적으로 모든 흡연 지표의 상대 위험도가 수도권에서 높은 경향을 보였다(Figure 1-3).

고 찰

본 연구에서는 지역사회 건강조사(KCHS) 자료를 이용하여 지역 단위 흡연 지표(흡연율, 가정/직장 내 간접 흡연율)과 네 가지 심혈관계 질환 유병률(30세 이상 고혈압, 50세 이상 뇌졸중, 40세

이상 심근경색, 40세 이상 협심증)의 연관관계를 베이지안 모형을 바탕으로 분석하였다. 그 결과 전국 단위에서 흡연율은 뇌졸중(RR=1.060)과 고혈압(RR=1.016)에 유의하게 연관되는 것을 확인하였으며, 간접 흡연 또한 뇌졸중(RR=1.013-1.037), 협심증(RR=1.006-1.016), 및 고혈압(RR=1.004-1.010)과 유의한 연관관계가 존재하였다. 그리고 가정 내 간접흡연 노출(RR=1.003-1.037)이 직장 내 간접흡연 노출(RR=0.998-1.013) 보다 심혈관계 질환 유병에 높은 연관성을 보였다.

그리고 본 연구에서는 전국 상대 위험도뿐 아니라 253개 지역별 위험도를 세부적으로 분석하였다. 이는 특별한 지역적 특성 (지리적 위치, 환경, 소득, 인구 구성 등)으로 인해, 지역별 유병률의 수준 및 지역별로 흡연이 심혈관계 질환 유병에 미치는 영향이 다를 것이라는 가정을 바탕으로 하였다. 분석 결과 지역에 따라 상이한 상대 위험도가 추정되었으며, 지역별로 흡연이 심혈관계 질환의 유병에 미치는 영향이 다르다는 것을 확인하였으며, 흡연 및 간접흡연의 영향이 모든 심혈관 질환에 걸쳐 도시-공업 지역에서 높은 것을 확인 할 수 있었다.

담배의 주요 성분인 니코틴은 중추와 말초에 작용하여 카테콜아민과 다른 신경 전달 물질들의 분비를 촉진하는 교감신경 자극물질이며, 심박수, 혈압 및 심박출량 상승과 같은 심혈관계 영향을 미친다. 뿐만 아니라 니코틴은 유리지방산 동원이 일어나게 하여 저밀도지단백 증가와 고밀도 지방의 감소를 초래하여, 혈관수축 작용을 증가시키고 혈관내피세포의 손상과 죽상동맥경화증의 진행을 가속화 시킨다 [1]. 또한 흡연 및 간접흡연으로 흡입 되는 일산화탄소는 헤모글로빈과 결합하여 저산소증을 일으키고 이는 적혈구의 양과 혈액 점성 증가를 야기하여 혈전 생성과 동맥경화에 직접적인 작용을 한다고 알려져 있다 [22]. 이러한 작용을 통하여 흡연은 관상동맥질환뿐만 아니라 동맥벽의 구조적 손상을 야기하며, 죽상동맥경화증으로 인한 허혈성 뇌졸중 및 동맥류의 생성과 파열로 인한 비외상성 지주막하 출혈과도 연관이 있다고 연구되어 있다 [23].

본 연구는 이러한 생물학적 기인과 상응하는 결과를 제시하고 있다. 연구 결과, 흡연 지표와 심근경색 및 협심증과는 통계적으로 유의한 관계를 보이지는 않았지만, 모든 심혈관 질환에서 전국 상대 위험도는 1 이상으로 추정되었다(RR=1.004-1.010). 이 결과는 미국과 노르웨이에서 실시된 흡연과 심장질환 관계에 관한 연구의 결과와도 상응한다[3, 4]. 한편 20년 동안 후향적 연구를 시행한 해외 연구에 따르면, 간접흡연에 대한 노출 정도(cotinine concentration)가 클수록 관상동맥질환과 뇌졸중 발생의 위험이 높아진다고 하였다[24]. 또 다른 연구에서는 실험-대조군 방법을 이용하여 간접 흡연 시 심근경색에 대한 위험도가 68% 증가함을 밝혀냈다[25]. 또한, 6개국 연구 결과를 메타분석으로 종합한 연구에서도 공통적으로 심혈관계 질환에 대한 상대위험도 값이 1.2와 1.3사이로 나타났다[26]. 한 연구에서는 담배의 끝에서 나오는 연기가 필터를 지난 연기보다 더 유해하기 때문에 간접흡연이 직접흡연보다 더 해롭다는 결과를 보였다[27]. 본 연구의 결과 또한 유사한 결과를 보였으며, 연구결과 협심증에서 가정 내 흡연(RR=1.016)이 직접흡연(RR=1.007) 보다 상대위험도의 값이 더 높게 추정되었다.

그럼에도 불구하고 본 연구에는 몇 가지 단점이 존재한다. 첫째로 이 연구는 오직 연관성을 분석하였을 뿐이지, 흡연 지표와 심혈관계 질환 유병률 사이의 인과관계를 분석한 것은 아니다. KCHS는 단면 조사 연구(Cross-sectional study)이기 때문에, 흡연 지표와 심혈관계 질환의 시간 관계를 고려하기가 매우 어렵다[18]. 또한 본 연구는 개인 수준의 위험 인자를 고려하지 못하였다. 그러므로 본 연구의 결과는 인과 관계가 아닌 지역 상관성으로 해석되어야 한다. 두 번째로는 흡연 및 간접 흡연에 취약한 지역은 추정할 수 있었지만, 취약의 생물학적/역학적 원인은 찾지 못하였다. 본 연구에서는 상대적으로 도시 지역(공업 지역 포함)이 흡연 및 간접에 심혈관 질환 유병이 취약한 것을 확인 할 수 있었으나, 성-연령이 보정된 상황에서 근본적인 가설 및 원인 제공은 하지 못하였다. 이어지는 다른 연구에서는 다양한 조사와 발전된 분석 방법을 이용하여, 위 결과를 설명할 수 있는 원인을 찾길 기대한다. 마지막으로, 본 연구는 사회적 지표를 고려하지 못하였다는 단점이 있다. 심혈관 질환은 소득 수준이나 환경 같은 사회적 요소에 영향을 받을 수 있는데, 본 연구에서는 자료의 한계로 인하여 사회적 지표를 적절하게 고려하지 못하였다. 다만 혼합 모형(Random effect model)을 이용한 종단(Longitudinal) 분석[28, 29]으로 잠재적인 지역별 특징을 고려함으로써, 사회적 지표를 고려하지 못한 단점을 일정 부분 보완하였다.

몇 가지 단점에도 불구하고, 본 연구는 큰 장점을 지니고 있다. 첫 번째로 150만 명 이상의 사람들을 대상으로 하여 심혈관계 질환과 흡연 지표의 상관성을 보았기 때문에, 높은 정확도의 연관성 추정이 가능했다. 특히 네 가지 심혈관 질환 중 대부분의 질환이 흡연 지표와 높은 연관성을 보였으며, 그 중 뇌졸중은 흡연 및 간접 흡연과 가장 큰 연관성을 보였다(RR=1.013-1.060). 두 번째로 발전된 통계적 방법을 사용하여, 전국 단위뿐만 아니라 지역 단위(시/군/구)의 상대 위험도까지 추정하였다. Integrated Nested Laplace Approximation (INLA) [20]는 베이지안 통계 분석에서 널리 사용되는 Markov Chain Monte Carlo (MCMC) 방법과 같이 사후 분포를 추정하기 위해 사용되는 계산 방법이지만, 라플라스 적분 근사를 사용하기 때문에 MCMC 보다 계산 속도가 빠르고, 분포 확장이 용이하다[30]. 본 연구는 이런 특징을 가진 INLA를 사용하여 지역상관성을 고려한 베이지안 계층화 분석을 실시함으로써 전국과 지역 단위의 위험도를 동시에 분석하였다는 장점을 가진다.

나아가 본 연구는 국민 건강을 위해 전국적으로 흡연 및 간접 흡연을 제한하는 공중 보건 정책의 근거로 사용될 수 있으며, 특히 심혈관계 질환에 취약할 수 있는 집단이 많은 지역의 지역 특화적 보건 정책 수립에 기여할 수 있다고 여겨진다. 즉, 노인 및 경제적 취약 계층과 같이 심혈관계 질환에 취약한 집단이 다수 상주하는 지역은, 더욱 적극적으로 흡연 제한 및 실내에서의 간접 흡연에 대한 대책을 마련할 필요가 있다.

감사의 글

본 연구는 환경부 “기후변화대응 환경기술개발사업(과제번호: 2014001310007)” 지원으로 수행되었음.

참고문헌

1. Bullen C. Impact of tobacco smoking and smoking cessation on cardiovascular risk and disease. Expert review of cardiovascular therapy. 2008; 6(6):883-95.
2. Control CfD, Prevention. Smoking-attributable mortality, years of potential life lost, and productivity losses--United States, 2000-2004. MMWR Morbidity and mortality weekly report. 2008;57(45):1226.
3. Kuller LH, Ockene JK, Meilahn E, Wentworth DV, Svendsen KH, Neaton JD, et al. Cigarette smoking and mortality. Preventive medicine. 1991; 20(5):638-54.
4. Håheim LL, Holme I, Hjermann I, Leren P. Risk factors of stroke incidence and mortality. A 12-year follow-up of the Oslo Study. Stroke. 1993; 24(10):1484-9.
5. Kannel WB, D'Agostino RB, Belanger AJ. Fibrinogen, cigarette smoking, and risk of cardiovascular disease: insights from the Framingham Study. American heart journal. 1987;113(4):1006-10.
6. Ockene IS, Miller NH. Cigarette smoking, cardiovascular disease, and stroke a statement for healthcare professionals from the American Heart Association. Circulation. 1997;96(9):3243-7.
7. Smoke T, Smoking I. IARC monographs on the evaluation of carcinogenic risks to humans. IARC, Lyon. 2004:1-1452.
8. Glantz SA, Parmley WW. Passive smoking and heart disease. Epidemiology, physiology, and biochemistry. Circulation. 1991;83(1):1-12.
9. Law MR, Morris J, Wald NJ. Environmental tobacco smoke exposure and ischaemic heart disease: an evaluation of the evidence. Bmj. 1997; 315(7114):973-80.
10. Thun M, Henley J, Apicella L. Epidemiologic studies of fatal and nonfatal cardiovascular disease and ETS exposure from spousal smoking. Environmental health perspectives. 1999;107(Suppl 6):841.
11. Lee EH, Park SK, Ko KP, Cho IS, Chang SH, Shin HR, et al. [Cigarette smoking and mortality in the Korean Multi-center Cancer Cohort (KMCC) study]. Journal of preventive medicine and public health=Yebang Uihakhoe chi. 2010;43(2):151-8.
12. Meng KH. Smoking-attributable mortality among Korean adults. Epidemiology and Health. 1988;10(2):138-45.
13. Jee SH, Lee JK, Kim IS. Smoking-attributable mortality among Korean adults: 1981-2003. Korean Journal of Epidemiology. 2006;28(1):92-9.
14. Jung KJ, Yun YD, Baek SJ, Jee SH, Kim IS. Smoking-attributable mortality among Korean adults, 2012. J Korean Soc Health Stat. 2013;28(28): 36-48.
15. Korea S. Korean statistical information service. Top. 2013;20:2006-11. http://kosis.kr/statHtml/statHtml.do?orgId=117&tblId=DT_H_SM&conn_path=I2&language=en
16. Kim J, Son M. National health insurance statistical yearbook. Seoul: Health Insurance Review and Assessment Service and National Health Insurance Service. 2014; 62-67.
17. Kim YT, Choi BY, Lee KO, Kim H, Chun JH, Kim SY, et al. Overview of Korean Community Health Survey. Journal of the Korean Medical Association/Taehan Uisa Hyophoe Chi. 2012;55(1).
18. Lee WH, Choo J-Y, Son J-Y, Kim H. Association between long-term exposure to air pollutants and prevalence of cardiovascular disease in 108 South Korean communities in 2008–2010: A cross-sectional study. Science of The Total Environment. 2016;565:271-8.
19. Besag J, York J, Mollié A. Bayesian image restoration, with two applications in spatial statistics. Annals of the institute of statistical mathematics. 1991;43(1):1-20.
20. Rue H, Martino S, Chopin N. Approximate Bayesian inference for latent Gaussian models by using integrated nested Laplace approximations. Journal of the royal statistical society: Series b (statistical methodology). 2009;71(2):319-92.
21. Martino S, Rue H. Implementing approximate Bayesian inference using Integrated Nested Laplace Approximation: A manual for the inla program. Department of Mathematical Sciences, NTNU, Norway. 2009.
22. Ambrose JA, Barua RS. The pathophysiology of cigarette smoking and cardiovascular disease: an update. Journal of the American college of cardiology. 2004;43(10):1731-7.
23. Kurth T, Kase CS, Berger K, Gaziano JM, Cook NR, Buring JE. Smoking and risk of hemorrhagic stroke in women. Stroke. 2003;34(12):2792-5.
24. Whincup PH, Gilg JA, Emberson JR, Jarvis MJ, Feyerabend C, Bryant A, et al. Passive smoking and risk of coronary heart disease and stroke: prospective study with cotinine measurement. Bmj. 2004;329(7459):200-5.
25. Wells AJ. Heart disease from passive smoking in the workplace. Journal of the American College of Cardiology. 1998;31(1):1-9.
26. He J, Vupputuri S, Allen K, Prerost MR, Hughes J, Whelton PK. Passive smoking and the risk of coronary heart disease—a meta-analysis of epidemiologic studies. New England Journal of Medicine. 1999;340(12): 920-6.
27. Dobson R. Smoke from cigarette tip is more toxic than main inhaled smoke. BMJ. 2005;331(7530):1425.
28. Laird NM, Ware JH. Random-effects models for longitudinal data. Biometrics. 1982:963-74.
29. Liang K-Y, Zeger SL. Longitudinal data analysis using generalized linear models. Biometrika. 1986;73(1):13-22.
30. Blangiardo M, Cameletti M. Spatial and spatio-temporal bayesian models with R-INLA: John Wiley & Sons; 2015.
